# Neurotransmitter regulatory networks in persistent postural-perceptual dizziness: potential mechanisms and therapeutic implications

**DOI:** 10.3389/fneur.2026.1921702

**Published:** 2026-09-08

**Authors:** Yu Zhu, Qi Han, Xin Teng, Tao Jing, Li Sun

**Affiliations:** 1 Chinese Medicine College, Changchun University of Chinese Medicine, Changchun, China; 2 Center for Dizziness and Balance Disorders, Jilin Academy of Chinese Medicine Sciences, Changchun, China; 3 Department of Neurology, Jilin Provincial Neuropsychiatric Hospital, Siping, China

**Keywords:** cognitive behavioral therapy, functional vestibular disorders, neurotransmitters, persistent postural-perceptual dizziness, transcranial magnetic stimulation, vestibular and balance rehabilitation therapy, vestibular compensation

## Abstract

Persistent postural-perceptual dizziness (PPPD) is a chronic functional vestibular disorder characterized by persistent non-spinning dizziness, subjective unsteadiness, and symptom exacerbation induced by upright posture, movement, and complex visual motion. Abnormal vestibular sensory reweighting, increased visual dependence, postural hypervigilance, and comorbid anxiety are considered to contribute to the persistence of symptoms. Neurotransmitter systems, including serotonin, norepinephrine, dopamine, gamma-aminobutyric acid, glutamate, histamine, acetylcholine and neuropeptides, may participate in the disease-related neural network imbalance by modulating vestibular signal processing, emotional responses, and central compensation. However, direct evidence for PPPD-specific neurotransmitter abnormalities remains limited. In treatment, selective serotonin reuptake inhibitors and serotonin–norepinephrine reuptake inhibitors are commonly employed in clinical practice, yet evidence from placebo-controlled randomized trials remains insufficient. Vestibular and balance rehabilitation therapy, cognitive behavioral therapy, and transcranial magnetic stimulation may be incorporated into comprehensive management; however, the level of evidence and long-term efficacy differ across interventions. Future studies should integrate clinical phenotypes, comorbidities, and neural network features to develop stratified diagnostic and therapeutic strategies, while also strengthening neurotransmitter-related imaging, biomarker discovery, and multicenter randomized controlled trials.

## 1 Introduction

Persistent postural-perceptual dizziness (PPPD) is a chronic functional vestibular disorder, with diagnostic criteria published by the Bárány Society in 2017 (1). A clinical study conducted in China found that patients were mainly aged 40–60 years and frequently experienced sleep disturbances and anxiety-related symptoms (2). At present, no validated objective diagnostic biomarker is available, and diagnosis therefore remains based on clinical criteria (1, 3). Evidence supporting disease-specific pharmacological treatment remains limited (4), and PPPD can substantially affect work and social functioning (5). Symptom persistence may be related to altered multisensory integration, visual dependence, and psychological and behavioral factors (1, 3, 6). Neurotransmitter networks may provide a biological framework for examining these mechanisms.

Neurotransmitters provide a chemical basis linking vestibular signal transmission, emotional regulation, and central compensation (7, 8). Serotonin (5-hydroxytryptamine, 5-HT), norepinephrine (NE), dopamine (DA), gamma-aminobutyric acid (GABA), glutamate (Glu), histamine, acetylcholine (ACh), and neuropeptides may participate in vestibular signal processing, regulation of vestibular nucleus excitability, postural control, anxiety responses, and neural plasticity (7, 8). Dysregulation of these systems may contribute to persistent dizziness, unsteadiness, and anxiety, although direct evidence of PPPD-specific neurotransmitter abnormalities remains limited. Pharmacological and non-pharmacological interventions relevant to these processes include selective serotonin reuptake inhibitors (SSRIs), serotonin–norepinephrine reuptake inhibitors (SNRIs), benzodiazepines (BZDs), vestibular and balance rehabilitation therapy (VBRT), and cognitive behavioral therapy (CBT) (4, 9).

Therefore, this narrative review examines the potential mechanisms of PPPD, current treatment evidence, and therapeutic implications from the perspective of neurotransmitter regulatory networks. It focuses on the roles of 5-HT, NE, DA, GABA, Glu, and histamine in vestibular function, emotional regulation, and vestibular–anxiety comorbidity. It also examines the mechanistic basis of relevant pharmacological and non-pharmacological interventions and identifies priorities for future mechanistic and clinical research.

## 2 Methods

To inform this narrative review, PubMed, Web of Science, and Google Scholar were searched without a lower date limit through June 2026. Search terms included combinations of “persistent postural-perceptual dizziness,” “chronic subjective dizziness,” and “phobic postural vertigo” with terms related to neurotransmitter systems and treatment, including serotonin or 5-hydroxytryptamine, norepinephrine or noradrenaline, dopamine, gamma-aminobutyric acid, glutamate, histamine, acetylcholine, neuropeptides, vestibular compensation, pharmacotherapy, vestibular and balance rehabilitation therapy, cognitive behavioral therapy, and transcranial magnetic stimulation. Reference lists of relevant consensus statements, systematic reviews, and primary studies were also examined to identify additional publications.

Titles and abstracts were reviewed for relevance to the mechanisms or treatment of PPPD, and full texts were examined when necessary. PPPD-specific human studies based on the Bárány Society diagnostic criteria and original mechanistic or clinical studies were prioritized. Evidence from chronic subjective dizziness and other predecessor syndromes, other vestibular disorders, animal models, and ex vivo studies was included when direct PPPD-specific evidence was unavailable and was identified in the synthesis as indirect evidence. Consensus documents and reviews were used mainly to provide definitions and background and to identify relevant primary studies. Because this was a narrative review, evidence was synthesized qualitatively; no formal risk-of-bias assessment, GRADE evaluation, or quantitative meta-analysis was performed.

## 3 Current research status of persistent postural-perceptual dizziness

### 3.1 Overview of persistent postural-perceptual dizziness

PPPD is defined by one or more symptoms of persistent dizziness, unsteadiness, or non-spinning vertigo that are present on most days for 3 months or more. Symptoms generally last for prolonged periods but may wax and wane in severity and need not be present continuously throughout the day; in many patients, they tend to increase as the day progresses (1). Among these symptoms, dizziness is primarily associated with disturbed spatial orientation and manifests as non-motion perceptual distortions. Postural unsteadiness often presents as a persistent sense of imbalance while standing or moving. Non-spinning vertigo may be perceived internally or externally: internal vertigo includes sensations of body swaying, rocking, or bobbing, whereas external vertigo involves an erroneous perception of movement or swaying of the surroundings (1). Symptom severity is closely related to specific environmental stimuli. Upright posture, active or passive movement, and exposure to moving or complex visual stimuli can exacerbate dizziness and unsteadiness (2, 10). Common provoking situations include standing, walking, riding in a vehicle, taking an elevator, viewing moving images, and exposure to complex visual backgrounds (2, 10). In particularly sensitive patients, symptoms may also worsen while sitting upright without support (1).

The onset of PPPD is frequently linked to acute, episodic, or chronic precipitating conditions. Common vestibular precipitants include benign paroxysmal positional vertigo, vestibular neuritis (VN), Ménière’s disease, and vestibular migraine (VM) (6, 10). Non-vestibular precipitants include panic attacks, concussion, whiplash injury, and other forms of psychological distress (6, 10). When PPPD develops in association with chronic conditions, such as generalized anxiety disorder or autonomic dysfunction, symptom onset may be gradual rather than clearly linked to a single acute event (10). Slowly progressive symptoms attributed to neurodegenerative disease require particular caution because an evolving neurological disorder may mimic, rather than precipitate, PPPD. Anxiety-related characteristics, including high neuroticism, state anxiety, hypervigilance, and negative illness cognitions, have been associated with the development or persistence of PPPD, although current evidence does not establish them as independent causal factors (6). Prospective studies of patients following acute VN have shown that visual dependence, autonomic arousal, and anxiety are more closely associated with persistent dizziness than the degree of residual peripheral vestibular dysfunction (11, 12). These findings suggest that a shift in central sensory weighting from adaptive vestibular compensation toward excessive visual dependence may contribute to persistent symptoms. Functional magnetic resonance imaging (fMRI) studies have also demonstrated altered activity or connectivity within visual, vestibular, spatial orientation, and attention-related networks in patients with PPPD (13–15). In particular, neuroticism-related changes in frontal attention-related and occipital visual regions may provide a potential link between anxiety-related personality traits, attention to visual motion, and visual dependence in PPPD (14).

### 3.2 Overview of the mechanisms of persistent postural-perceptual dizziness

The pathophysiological mechanisms underlying PPPD remain incompletely understood (16). The prevailing model proposes that, following an acute or chronic balance-disrupting event, initially adaptive postural and perceptual strategies may persist and become maladaptive, rather than reflecting persistent damage to the peripheral vestibular system alone (1, 16). In patients with persistent dizziness after acute vestibular neuritis, visual dependence, autonomic arousal, and anxiety during the acute phase have been more closely associated with subsequent symptoms than the degree of residual peripheral vestibular dysfunction (11, 12). After the precipitating condition has resolved or stabilized, altered sensory reweighting may persist, with excessive reliance on visual information relative to vestibular and somatosensory cues (1, 17). Posturographic studies have demonstrated impaired postural control across multiple sensory conditions and altered postural strategies in complex visual and cognitive environments in patients with PPPD (17, 18). Task-based neuroimaging studies have identified altered responses within visual, vestibular, and multisensory cortical regions during visual-motion or vestibular stimulation (13, 19). Resting-state functional magnetic resonance imaging has also demonstrated altered intra- and inter-network functional connectivity involving visual and other large-scale brain networks (20). These abnormalities may contribute to increased visual dependence and heightened sensitivity to self-motion or environmental motion (13, 19). However, these findings primarily demonstrate associations and should not be interpreted as establishing a single causal mechanism.

Beyond these pathophysiological processes, PPPD has multidimensional psychosocial consequences. In a qualitative study of working-age adults, patients described difficulty obtaining recognition and validation of their symptoms, disruption of self-identity, uncertainty regarding their ability to cope, and substantial effects on their personal, occupational, and social lives (5). A subsequent qualitative study similarly identified experiences of dismissal or non-belief, loss of identity, impaired psychological well-being, and difficulty making sense of persistent symptoms (21). These findings indicate that the psychosocial consequences of PPPD should be considered alongside vestibular symptoms in individualized, multidisciplinary care (5, 21).

## 4 Potential mechanisms of neurotransmitter regulatory networks in persistent postural-perceptual dizziness

To date, the neurotransmitter mechanisms underlying PPPD have not been directly confirmed. Existing evidence primarily stems from basic vestibular pharmacology, animal experiments, ex vivo preparations, studies of other vestibular disorders, chronic dizziness research, and investigations related to anxiety or depression. These findings offer biological plausibility for understanding the persistence of PPPD symptoms, abnormal vestibular compensation, increased visual dependence, and the comorbidity of vestibular disorders with anxiety. However, they cannot be directly equated with neurotransmitter abnormalities specific to PPPD. Consequently, this section delineates a three-tiered evidence framework: first, it summarizes fundamental pharmacological findings in the vestibular system; second, it elucidates how these findings may provide mechanistic insights into the maintenance of PPPD symptoms; and third, it underscores the absence of direct evidence for PPPD-specific alterations in neurotransmitter levels, receptor expression, or neurotransmitter network function.

### 4.1 Overview of the neurotransmitter basis of the vestibular system

The peripheral sensory organs of the vestibular system comprise three semicircular canals, the saccule, and the utricle. Hair cells transduce mechanical stimuli—such as angular and linear accelerations of the head—into neural signals, which are subsequently transmitted via vestibular afferent fibers to the central vestibular pathways. At peripheral vestibular synapses, Glu serves as the primary excitatory neurotransmitter released by hair cells, facilitating rapid signal transmission between hair cells and primary vestibular afferent neurons (22, 23). The vestibular efferent system primarily originates in the brainstem (24), with its cholinergic fibers projecting to vestibular hair cells and afferent nerve terminals. ACh thereby modulates peripheral vestibular input through nicotinic and muscarinic acetylcholine receptors (25, 26). In addition to Glu and ACh, GABAergic neurons are present in the central vestibular nuclei (27), whereas histamine receptors, substance P, calcitonin gene-related peptide (CGRP), and opioid peptides have been identified in peripheral or central vestibular structures (28–30). These substances can act as neuromodulators, influencing vestibular neuronal excitability and synaptic transmission through their corresponding receptors (31, 32). They may consequently alter transmitter release, receptor responses, and neuronal discharge characteristics. Thus, multi-neurotransmitter regulation within the peripheral and central vestibular pathways provides a cellular and receptor basis for the effects of pharmacological agents on vestibular information transmission. However, this foundational evidence should not be directly equated with specific neurotransmitter abnormalities in patients with PPPD.

**Figure 1:**
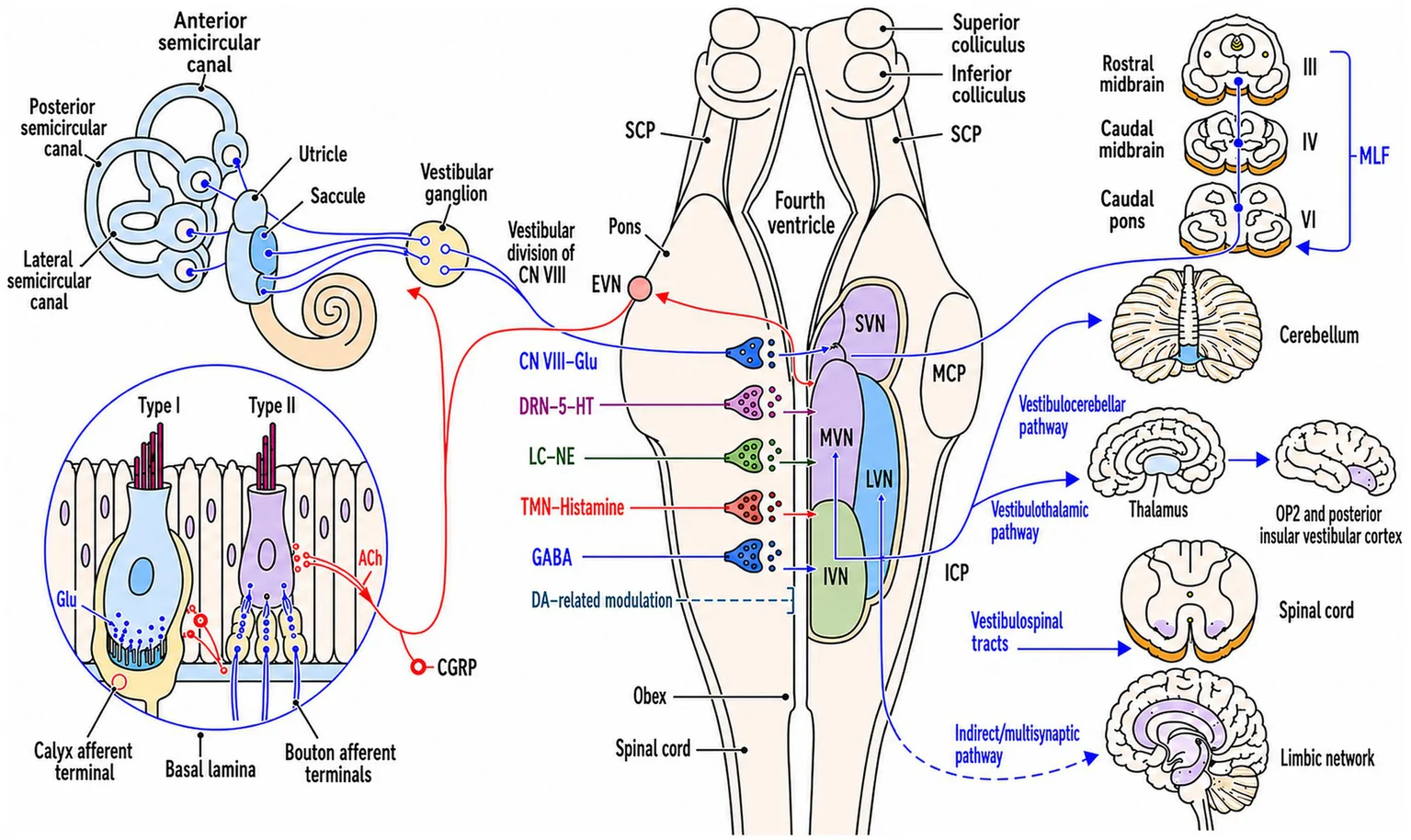
Peripheral and central vestibular pathways and representative neurotransmitter-related modulation. The schematic illustrates the peripheral vestibular end organs, type I and type II vestibular hair cells and their afferent and efferent innervation, the vestibular nuclear complex, and major ascending and descending projections. Glutamate (Glu) is depicted as the principal excitatory neurotransmitter released at vestibular hair cell–afferent synapses, whereas acetylcholine (ACh) is depicted as the principal neurotransmitter of the vestibular efferent system. Representative influences associated with 5-hydroxytryptamine (5-HT), norepinephrine (NE), histamine, and gamma-aminobutyric acid (GABA) on the vestibular nuclei are also shown. Dopamine (DA) is presented as indirect or pathway-unspecified modulation because direct pathway-level evidence remains limited. Calcitonin gene-related peptide (CGRP) is shown as a representative neuropeptide associated with vestibular efferent signaling. Substance P and enkephalin are discussed in the text as species-dependent or putative neuropeptide modulators and are not depicted as established pathways. This schematic does not imply persistent postural-perceptual dizziness-specific neurotransmitter abnormalities. ACh, acetylcholine; CGRP, calcitonin gene-related peptide; CN VIII, cranial nerve VIII (vestibulocochlear nerve); DA, dopamine; DRN, dorsal raphe nucleus; EVN, efferent vestibular nucleus; GABA, gamma-aminobutyric acid; Glu, glutamate; ICP, inferior cerebellar peduncle; IVN, inferior vestibular nucleus; LC, locus coeruleus; LVN, lateral vestibular nucleus; MCP, middle cerebellar peduncle; MLF, medial longitudinal fasciculus; MVN, medial vestibular nucleus; NE, norepinephrine; OP2, cytoarchitectonic area OP2 of the parietal operculum; PPPD, persistent postural-perceptual dizziness; SCP, superior cerebellar peduncle; SVN, superior vestibular nucleus; TMN, tuberomammillary nucleus; 5-HT, 5-hydroxytryptamine. III, oculomotor nucleus; IV, trochlear nucleus; VI, abducens nucleus.

Figure 1 summarizes the peripheral and central vestibular pathways and the representative neurotransmitter-related modulation discussed in this section.

Central monoamine neurotransmitters, including DA, NE, and 5-HT, serve as crucial signaling molecules in the central nervous system. They contribute to emotional regulation (33), motor control (34), and cognitive function (35). Their signaling is dynamically regulated through biosynthesis, synaptic release, reuptake, and metabolic inactivation (36, 37). Neurotransmission occurs at synapses—the junctions between neurons—where electrical signals are converted into chemical signals in the form of neurotransmitters. Action potentials propagate along presynaptic neurons, triggering the release of these molecules into the synaptic cleft, where they interact with receptors on the membranes of postsynaptic neurons (38). For many neurotransmitters, signal transmission is terminated by reuptake through integral membrane transporters located on presynaptic neurons and glial cells (39). The solute carrier family 6 (SLC6), also known as the neurotransmitter:sodium symporter family, includes members that transport monoamines (40–42), GABA (43), glycine (44), and osmoregulatory substances such as betaine (45). SLC6 transporters couple substrate uptake to the movement of Na^+^ and, for many family members, Cl^−^ down their electrochemical gradients. This energetically favorable downhill ion movement provides the driving force for substrate accumulation against its concentration gradient—an energetically unfavorable “uphill” process that would not proceed spontaneously without ion coupling (46). Pathogenic dysfunction of individual SLC6 transporters has been associated with specific disorders, including epilepsy (47) and orthostatic intolerance with tachycardia (48).

#### 4.1.1 Serotonergic regulation of vestibular and emotional processing

5-HT is an endogenous indoleamine monoamine neurotransmitter that exerts neuromodulatory effects in the central nervous system (49). Through distinct 5-HT receptor subtypes, it can exert inhibitory or excitatory control over cortical and limbic networks (50, 51), participating in emotional regulation (33), cognitive function (52), and stress responses (49). Altered serotonergic function has been associated with anxiety and depressive disorders (53, 54). The biosynthesis of 5-HT begins with the essential amino acid tryptophan (Trp), which is catalyzed by tryptophan hydroxylase to form the intermediate 5-hydroxytryptophan; this is subsequently decarboxylated by aromatic L-amino acid decarboxylase to form 5-HT (55). Therefore, the transport of Trp across the blood–brain barrier (56) and its availability in the brain (57) are important factors influencing central 5-HT synthesis. A clinical experimental study showed that Trp depletion increased motion sickness-related dizziness, nausea, and the illusion of movement in healthy controls to levels approaching those observed in migraineurs (58), suggesting that reduced serotonergic activity may increase susceptibility to vestibulo-ocular disturbances during motion sickness.

Experimental studies have shown that 5-HT modulates synaptic transmission in the medial vestibular nucleus and vestibular-related motor function (59), as well as the vestibulo-ocular reflex (60). A subpopulation of serotonergic neurons in the dorsal raphe nucleus (DRN) sends collateral projections to both the vestibular nuclei and the central amygdaloid nucleus, providing an anatomical substrate for the coordinated modulation of vestibular and affective processing (61). In a rat model of intratympanic gentamicin-induced vestibular impairment, transient anxiety-like behavior was accompanied by region-specific monoaminergic changes: NE and the 5-HT metabolite 5-hydroxyindoleacetic acid increased in the medial vestibular nucleus, whereas NE and 5-HT increased in the locus coeruleus (LC) and DRN (62). However, during the interictal period of VM, persistent dizziness and unsteadiness should be assessed according to both the VM and PPPD diagnostic criteria (1, 63). Clinical cohort studies also indicate that migraine or VM and anxiety disorders may coexist with persistent functional dizziness or PPPD and should be differentiated during clinical assessment (10, 64). Small case–control studies have reported an association between rs770963777 in the *HTR6* gene and VM (65), and between dopamine receptor D2 gene (*DRD2*) TaqIA and PPPD (66). Overall, these findings suggest that monoaminergic systems may contribute to vestibular and affective features relevant to VM and PPPD; however, they do not establish a shared neurobiological basis for VM–PPPD comorbidity. These findings may provide mechanistic clues to the maintenance of PPPD symptoms, but direct evidence of PPPD-specific abnormalities in 5-HT levels, receptor expression, or network function remains lacking.

#### 4.1.2 Noradrenergic modulation of vestibular function and stress responses

The inferior vestibular nucleus is an important brainstem center involved in postural control and balance. In rat brainstem slices, NE directly regulated neuronal activity in the inferior vestibular nucleus and produced a net excitatory effect, suggesting that the noradrenergic system may participate in the modulation of vestibular reflexes and postural control (67). In a rat model of repetitive blast-induced traumatic brain injury, rotarod performance was significantly impaired. NE levels were increased in the LC, whereas NE and DA levels were reduced in the vestibular nuclei; 5-HT levels were increased in the motor cortex and lumbar spinal cord (68). These regional monoaminergic changes occurred alongside impaired balance and motor performance, but the study did not establish a causal relationship. In a clinical study of chronic subjective dizziness (CSD), one of the historical precursor syndromes incorporated into the PPPD construct, serum cortisol and epinephrine levels were reported to be elevated, whereas serum serotonin levels were lower than those in healthy controls (69). Collectively, these findings provide indirect mechanistic clues from experimental injury and CSD but do not establish monoaminergic abnormalities as direct causes of dizziness or unsteadiness in PPPD. Direct evidence of PPPD-specific abnormalities in NE levels, NE receptor expression, or NE-related network function remains lacking.

#### 4.1.3 Dopaminergic signaling in emotion and motor control: candidate links to PPPD susceptibility

DA and dopaminergic circuits in the brain contribute to reward and motor control (70), motivation (71), and anxiety-related behavior (72). These functions may provide partial biological context for postural hypervigilance, avoidance behavior, and comorbid anxiety in PPPD; however, their specific relevance to these PPPD features remains hypothetical because these studies did not investigate PPPD. Outside the brain, DA is involved in signaling in the eye, cardiovascular system, and endocrine pancreas (73). Dysregulated dopaminergic signaling is implicated in several psychiatric disorders (74), and some illicit psychostimulants and prescription medications, including those used to treat attention-deficit/hyperactivity disorder, can modulate dopaminergic and noradrenergic signaling by affecting DA and NE transporters (75). Some drugs with DA receptor antagonist properties are used for vertigo-related nausea or vomiting (8), but there is no evidence supporting DA-targeted drugs as standard therapy for PPPD. DA should therefore be discussed as indirect biological evidence and a candidate research direction rather than as an established PPPD pathogenic mechanism or mature therapeutic target.

Genetic research on PPPD remains limited. The *DRD2*/*ANKK1* TaqIA polymorphism (rs1800497) has been investigated as a candidate genetic factor. A small case–control study reported that the A1 allele was more frequent in patients with PPPD than in controls, suggesting that it may represent a susceptibility factor for PPPD (66). In a positron emission tomography study of healthy volunteers, the A1 allele was associated with reduced striatal D2 receptor availability (76). However, whether these associations contribute to vulnerability to PPPD after an acute peripheral vestibular disorder or another precipitating event remains unknown. Neuroticism is significantly associated with anxiety disorders (77), and elevated neuroticism, state anxiety, and body vigilance have been investigated as potential risk factors for PPPD (6). Anxiety disorders and VM are also common precipitating or comorbid conditions in PPPD (10), and patients with VM have higher anxiety levels than patients with migraine without vestibular symptoms (78). At the neurotransmitter level, alterations in serotonergic and dopaminergic systems have been implicated in anxiety-related disorders (79, 80); however, this evidence is not specific to PPPD. A case–control study also reported an association between the rs770963777 polymorphism in the *HTR6* gene, and VM (65). Larger multicenter and functional studies are required to confirm these candidate-gene associations. These findings may offer preliminary mechanistic clues to PPPD susceptibility; however, direct evidence of PPPD-specific abnormalities in DA levels, D2 receptor expression or availability, or DA-related network function remains lacking.

#### 4.1.4 Gamma-aminobutyric acid signaling in vestibular processing and compensation

GABA is an important inhibitory neurotransmitter in the vestibular nuclei. GABA_A_ and GABA_B_ receptors play key roles in the coordination of central vestibular pathways (81), and GABAergic pathways contribute to commissural inhibition between the bilateral vestibular nuclear complexes (VNCs) (81, 82); GABAergic neurons in the vestibular nuclei are involved in various aspects of vestibular sensory processing and are associated with balance maintenance, postural control, and gaze stability (27, 82). GABA is the primary inhibitory neurotransmitter in the central nervous system of mature mammals (83). Abnormal regulation of GABAergic inhibition within the vestibular nuclei may disrupt the excitatory-inhibitory balance between the bilateral vestibular nuclei and affect vestibular compensation (81), contributing to reduced visual stability, postural instability, and symptoms of vertigo or dizziness through the vestibulo-ocular and vestibulospinal reflexes (8, 84).

The mechanisms of GABA action in the vestibular system depend on receptor subtype. Activation of presynaptic GABA_B_ receptors can reduce neurotransmitter release in vestibular nucleus neurons (85). The vestibular nuclei are known to receive strong inhibitory inputs mediated by GABA_A_ and GABA_B_ receptors (81). These GABAergic inputs include commissural projections between the vestibular nuclei and inputs from other brainstem regions (82, 86). BZDs enhance GABAergic inhibition through positive allosteric modulation of GABA_A_ receptors (8). However, current evidence does not support their routine use for acute vertigo (87), and they are not recommended as routine long-term treatment for PPPD (88). These findings may provide mechanistic insights into the maintenance of PPPD symptoms; however, there is currently a lack of direct evidence regarding PPPD-specific abnormalities in GABA levels, GABA receptor expression, or GABAergic networks.

#### 4.1.5 Glutamatergic transmission in vestibular and reflex pathways

In the vestibular system, Glu is an excitatory amino acid (89) and the primary neurotransmitter released by vestibular hair cells (22, 23). Glu acts on ionotropic glutamate receptors (90) and metabotropic glutamate receptors (mGluRs) (91). Ionotropic Glu receptors are located at synapses between vestibular hair cells and primary afferent neurons and mediate input signals from vestibular hair cells (23, 90). mGluRs participate in synaptic transmission and plasticity between primary afferent neurons and secondary vestibular nucleus neurons (91).

Experimental studies have shown that excitatory synaptic currents mediated by Glu receptors activate afferent nerve fibers innervating vestibular hair cells, supporting Glu as the primary afferent neurotransmitter released by vestibular hair cells (23). In the peripheral vestibular end organs, afferent synapses between hair cells and primary afferent neurons involve glutamatergic transmission (23, 92). In the central vestibular pathways, glutamatergic neurotransmission is also present in the vestibular nuclei and their associated projection pathways (91, 93) and contributes to the vestibulo-ocular and vestibulospinal reflexes (7), as well as the vestibulo-autonomic reflex (93). The balance between Glu and GABA contributes to the stability of vestibular signaling (94). These findings may provide mechanistic insights into the maintenance of PPPD symptoms; however, direct evidence of PPPD-specific abnormalities in Glu or GABA levels or related neurotransmitter networks remains insufficient.

#### 4.1.6 Histaminergic regulation of vestibular excitability and compensation

Histamine is an important modulatory biogenic amine in the vestibular system. Its H1, H2, H3, and H4 receptors are G protein-coupled receptors that regulate neuronal excitability and neurotransmitter release through distinct intracellular signaling pathways (95). All four histamine receptor subtypes have been detected in the mouse inner ear and vestibular-related structures (28), suggesting that histamine signaling may be involved in peripheral vestibular sensory transmission and vestibular ganglion function. In the peripheral vestibular pathway, H3 receptors are expressed in calyx and dimorphic neurons of the mouse Scarpa ganglion (96). H4 receptor antagonists inhibit the discharge activity of primary vestibular neurons in rats, suggesting that H3 and H4 receptors may regulate vestibular afferent excitability (31). In the central vestibular pathway, histamine enhances the excitability of rat medial vestibular nucleus neurons through mechanisms involving both H1 and H2 receptors (97). Betahistine has weak H1 receptor agonist and H3 receptor antagonist activity (98). Its effects on medial vestibular nucleus neurons suggest that histaminergic regulation may influence vestibular nucleus excitability and may be relevant to the actions of drugs used for vertigo and motion sickness (97). Animal studies have also shown that H1 receptors contribute to vestibular compensation after unilateral vestibular lesions, suggesting that histaminergic signaling may be involved in central adaptation following vestibular injury (99).

Histamine may indirectly influence sensory reweighting and symptom persistence following vestibular precipitating events in PPPD by modulating peripheral vestibular afferent excitability (31, 96), vestibular nucleus neuron activity (97), and vestibular compensation (99). However, no studies have directly demonstrated PPPD-specific abnormalities in histamine levels, histamine receptor expression, or histaminergic pathways. Therefore, histamine should be considered a possible indirect contributor to PPPD mechanisms rather than a confirmed cause of the disorder or an established therapeutic target for PPPD.

#### 4.1.7 Acetylcholine and neuropeptides in vestibular modulation

ACh is a key neurotransmitter in the vestibular efferent system (100). Vestibular efferent neurons originate in the brainstem and project via the vestibular nerve to the peripheral vestibular sensory epithelium, hair cells, and vestibular afferent terminals (101). Vestibular efferent cholinergic signaling can modulate the excitability of hair cells and afferent terminals through nicotinic and muscarinic ACh receptors (25, 100). Animal studies have shown that nicotinic ACh receptors mediate efferent-induced excitation in calyx-bearing vestibular afferents (102). Therefore, ACh is more appropriately described as the principal neurotransmitter of the vestibular efferent system rather than the primary excitatory neurotransmitter at vestibular hair cell-afferent synapses (7, 100).

Neuropeptides may be involved in the regulation of peripheral and central vestibular pathways, but their roles are primarily modulatory (7, 103). CGRP- and substance P-like immunoreactivities have been observed in rat vestibular end organs, suggesting that both may participate in peripheral vestibular sensory modulation (29). CGRP has also been detected in the vestibular efferent nucleus and vestibular nuclei of rats. Its immunoreactivity increases following rotary stimulation, suggesting that CGRP may participate in responses to vestibular stimulation (104). Opioid signaling components are also present in the vestibular system. mRNA encoding preproenkephalin, the precursor protein from which enkephalin opioid peptides are generated, has been detected in gerbil vestibular efferent neurons projecting to the inner ear (30). μ-Opioid receptors in cultured rat vestibular afferent neurons modulate calcium currents and action potential firing and may thereby influence neuronal excitability and neurotransmitter release (32).

These studies suggest that ACh, CGRP, substance P, and opioid peptides may provide a neuropharmacological basis for understanding vestibular signal modulation and vestibular efferent control. However, this evidence primarily comes from animal experiments, *in vitro* studies, and basic vestibular pharmacology and does not establish PPPD-specific abnormalities in ACh or neuropeptides. PPPD is diagnosed according to clinical symptoms, symptom duration, exacerbating factors, and precipitating conditions (1); no diagnostic biomarkers based on ACh or neuropeptides have been established. Pharmacological evidence for PPPD remains limited (4), and no clinical evidence currently establishes ACh- or neuropeptide-targeted drugs as treatments for PPPD. Therefore, ACh and neuropeptides should be discussed as candidate regulatory mechanisms and future research directions rather than established PPPD-specific pathogenic mechanisms.

### 4.2 Mechanisms of vestibular-anxiety comorbidity

Previous neuroanatomical studies have identified connections between the VNC and the DRN, LC, and parabrachial nucleus (PBN), which participate in arousal and affective regulation (105, 106). In rats, unilateral labyrinthectomy produces dynamic monoaminergic changes in the medial vestibular nucleus (MVN): 5-HT and NE levels increase during the first 4 days, whereas DA levels increase at several postoperative time points (107). These findings support monoaminergic involvement in early vestibular compensation but do not establish uniform activation of the VNC-LC, VNC-DRN, and VNC-PBN pathways or directly explain anxiety-like behavior after vestibular injury. Animal studies have shown that 5-HT produces excitatory and inhibitory changes in the firing of rat vestibular nucleus neurons (108–110). 5-HT1A receptor-mediated attenuation of synaptic transmission in the MVN influences vestibular-related motor function relevant to posture and balance (59), and serotonin also modulates the amplitude of the vestibulo-ocular reflex in rats (60). Serotonergic and nonserotonergic neurons in the DRN send collateralized projections to both the vestibular nuclei and the central amygdala, providing an anatomical basis for interactions between vestibular and anxiety-related circuits (61).

NE is closely associated with vigilance, arousal, and stress responses, and the LC is its principal central source (111, 112). Experimental manipulation of LC-NE neurons in mice showed that increased tonic activity induces anxiety-like behavior, whereas inhibition during stress prevents subsequent anxiety-like behavior (113). Anatomical studies have demonstrated region-selective noradrenergic projections from the LC to the VNC (114). Reciprocal vestibular influences on LC activity and modulation of vestibular reflex gain have been proposed, but direct evidence remains limited (106, 115, 116). Evidence for the involvement of NE in vestibular compensation also remains limited. In rats, unilateral labyrinthectomy produces short-term, region-dependent increases in NE content across forebrain and cerebellar structures, suggesting noradrenergic involvement in early vestibular compensation (117). Studies using dopaminergic agents indicate that DA signaling modulates vestibular compensation after unilateral labyrinthectomy (118). In aged rats, D2 receptor agonism accelerated the recovery of spontaneous nystagmus and improved ambulation and motor coordination, whereas D2 receptor antagonism produced opposite effects (119).

**Figure 2:**
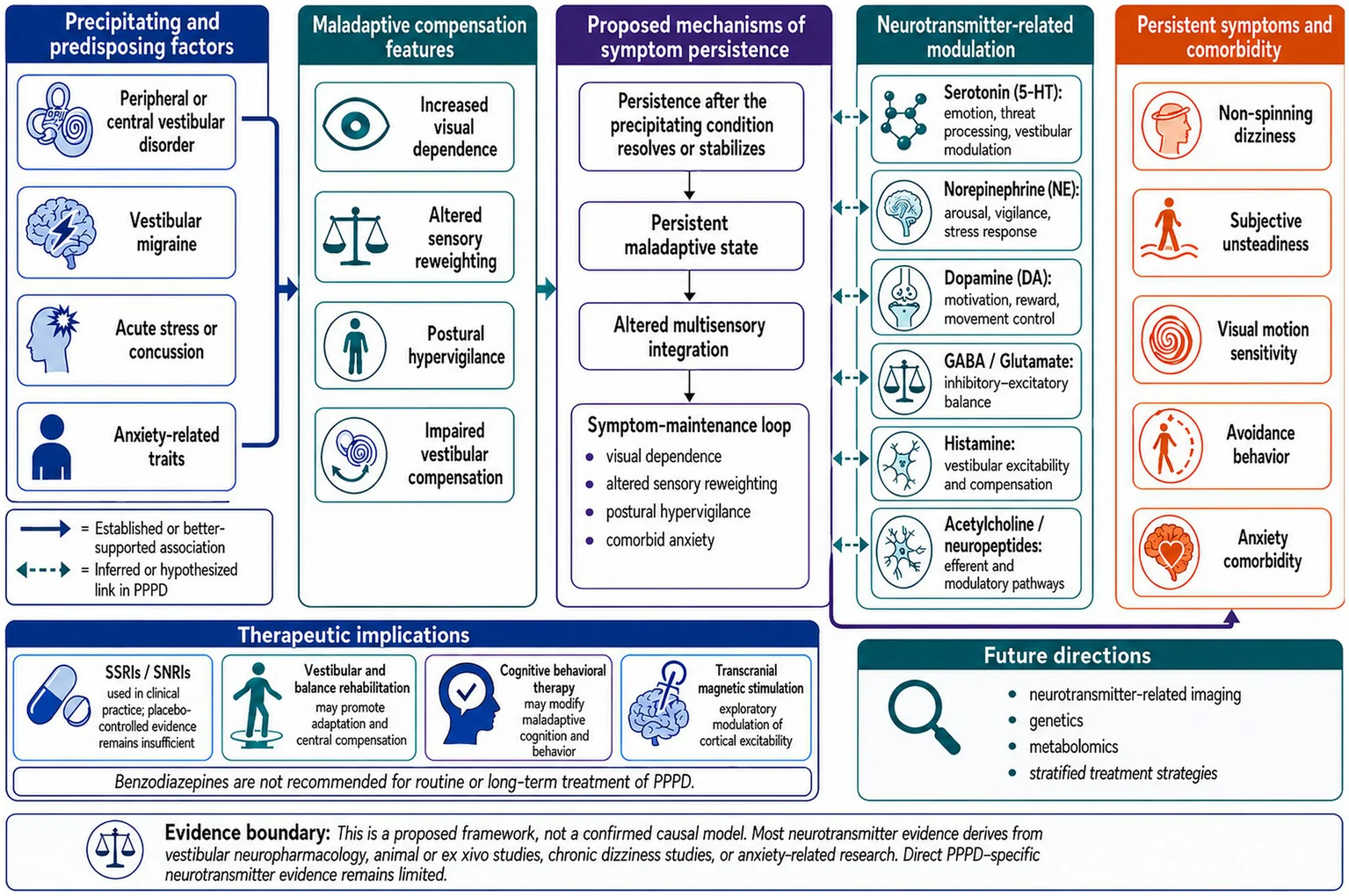
Proposed integrative framework for symptom persistence and neurotransmitter-related modulation in persistent postural-perceptual dizziness. Precipitating and predisposing factors are linked to maladaptive compensation features, including increased visual dependence, altered sensory reweighting, postural hypervigilance, and impaired vestibular compensation. Persistence after the precipitating condition resolves or stabilizes is depicted as a proposed maladaptive state involving altered multisensory integration and a symptom-maintenance loop. Representative neurotransmitter-related influences include serotonin, norepinephrine, dopamine, gamma-aminobutyric acid, glutamate, histamine, acetylcholine, and neuropeptides. The proposed persistence framework is linked to non-spinning dizziness, subjective unsteadiness, visual motion sensitivity, avoidance behavior, and anxiety comorbidity. Solid arrows indicate established or better-supported associations, whereas dashed arrows indicate inferred or hypothesized links in PPPD. The lower panels summarize selective serotonin reuptake inhibitors and serotonin–norepinephrine reuptake inhibitors, vestibular and balance rehabilitation, cognitive behavioral therapy, transcranial magnetic stimulation, the limitations of benzodiazepines, and future research directions. This schematic represents a proposed framework rather than a confirmed causal model, and direct PPPD-specific neurotransmitter evidence remains limited. 5-HT, 5-hydroxytryptamine; ACh, acetylcholine; DA, dopamine; GABA, gamma-aminobutyric acid; NE, norepinephrine; PPPD, persistent postural-perceptual dizziness; SNRIs, serotonin–norepinephrine reuptake inhibitors; SSRIs, selective serotonin reuptake inhibitors.

Taken together, these findings suggest that monoaminergic modulation of vestibular and emotion-related circuits may contribute to symptom persistence in PPPD. However, the current evidence is indirect and derives mainly from animal and mechanistic studies rather than direct investigations of PPPD. Figure 2 presents a proposed integrative framework linking precipitating and predisposing factors, maladaptive compensation features, representative neurotransmitter-related modulation, proposed mechanisms of symptom persistence, and persistent symptoms and comorbidity. The framework also summarizes potential therapeutic implications and future research directions and does not represent a confirmed causal model or imply established PPPD-specific neurotransmitter abnormalities.

Table 1 summarizes the neurotransmitter systems relevant to PPPD and the boundaries of current evidence.

**Table 1:** Neurotransmitter systems relevant to persistent postural-perceptual dizziness and their evidence boundaries.

Neurotransmitter system	Vestibular or neurobiological evidence	Potential relevance to PPPD	Direct PPPD-specific evidence	Potential clinical implications	Key references
Serotonin	5-HT can modulate cortical and limbic networks, vestibular nuclear activity, vestibular-related motor function, and anxiety-related circuits.	It may contribute to vestibular–emotional network imbalance, visual motion sensitivity, anxiety comorbidity, and symptom persistence.	No direct PPPD-specific evidence has demonstrated alterations in 5-HT levels, receptor expression, or network function.	The serotonergic system may provide mechanistic clues to vestibular–emotional interactions, anxiety comorbidity, and visual motion sensitivity; however, current evidence does not establish a shared serotonergic basis for VM and PPPD. It also provides a rationale for future studies using 5-HT-related neuroimaging, vestibular stimulation paradigms, and symptom-based phenotyping.	(33, 49–65, 108–110)
Norepinephrine	The LC projects to the vestibular nuclei and is involved in arousal, vigilance, stress responses, and regulation of vestibular reflexes.	It may contribute to postural hypervigilance, arousal-related symptom amplification, and vestibular–anxiety interactions.	No direct PPPD-specific evidence has demonstrated abnormalities in NE levels, receptor expression, or NE-related network function.	These findings support assessment of arousal, vigilance, stress responses, and vestibular–anxiety interactions in PPPD. The LC–NE system and its connections with the vestibular nuclear complex may be relevant targets for future studies of neural circuits, vestibular compensation, and clinical phenotyping.	(67–69, 111–117)
Dopamine	Dopaminergic circuits are involved in reward, motivation, motor control, neuroticism-related personality traits, and vestibular compensation in animal models.	They may provide a partial biological basis for avoidance behavior, movement-related vigilance, and susceptible phenotypes.	Limited. An association with the *DRD2*/*ANKK1* TaqIA polymorphism has been reported, but this does not establish dopaminergic dysfunction in PPPD.	This pathway may serve as a candidate mechanism for genetic susceptibility and clinical phenotyping, but it is not currently an established therapeutic target.	(66, 70–80, 118, 119)
Gamma-aminobutyric acid	GABA is the principal inhibitory neurotransmitter in the mature mammalian central nervous system and participates in commissural inhibition between vestibular nuclei.	Abnormal inhibitory regulation may affect vestibular compensation, the excitatory–inhibitory balance between bilateral vestibular nuclei, and postural stability.	No direct PPPD-specific evidence has established abnormalities in GABA levels, receptor expression, or network function.	This mechanism may explain the short-term vestibular suppressant effects of benzodiazepines, but it does not support their use as long-term therapy for PPPD.	(27, 81–88)
Glutamate	Glu is an excitatory amino acid and the principal neurotransmitter released by vestibular sensory hair cells. It acts on both ionotropic and metabotropic glutamate receptors.	Altered excitatory–inhibitory balance may affect vestibular signal processing and central compensation.	No direct PPPD-specific glutamatergic abnormalities have been demonstrated.	The balance between Glu and GABA may be relevant to vestibular signal stability and may provide mechanistic clues to symptom persistence in PPPD.	(22, 23, 89–94)
Histamine	Histamine receptors are expressed in the inner ear and vestibular pathways, and histamine can regulate vestibular nuclear excitability and vestibular compensation.	Histamine may influence vestibular excitability, compensation after vestibular events, and sensory reweighting.	No direct PPPD-specific histaminergic abnormalities have been demonstrated.	Histamine-related mechanisms are relevant to acute vestibular symptom control and future mechanistic research, but they do not support histamine pathways as long-term therapeutic targets for PPPD.	(28, 31, 95–99)
Acetylcholine	Acetylcholine is an important transmitter of the vestibular efferent system and can modulate hair cells and vestibular afferent terminals.	It may participate in vestibular efferent modulation and regulation of peripheral vestibular input.	No direct PPPD-specific evidence of acetylcholine-related abnormalities or therapeutic effects is currently available.	This pathway mainly helps explain the physiological mechanisms of the vestibular efferent system.	(7, 24–26, 100–102)
Neuropeptides	Neuropeptides may modulate peripheral and central vestibular pathways, but their effects are largely modulatory.	They may participate in vestibular sensory modulation, motion stimulus-related responses, and vestibular compensation.	No direct PPPD-specific evidence is currently available; existing findings are derived mainly from animal or ex vivo studies.	Neuropeptides should be regarded as an exploratory research direction rather than current diagnostic or therapeutic targets.	(7, 29, 30, 32, 103, 104)

PPPD, persistent postural-perceptual dizziness; 5-HT, 5-hydroxytryptamine; VM, vestibular migraine; NE, norepinephrine; LC, locus coeruleus; *DRD2*, dopamine receptor D2; GABA, gamma-aminobutyric acid; Glu, glutamate; ACh, acetylcholine; CGRP, calcitonin gene-related peptide.

## 5 Current status of treatment for persistent postural-perceptual dizziness

Currently, there is no standardized first-line treatment for PPPD, and clinical practice often depends on a comprehensive management approach (4, 120). Available interventions include pharmacotherapy, CBT, VBRT, and transcranial magnetic stimulation (TMS). Pharmacotherapy is used in clinical practice (121), but high-quality placebo-controlled evidence remains insufficient (4). A randomized study and a meta-analysis suggest that CBT may be beneficial as an adjunctive treatment (122, 123). Clinical studies have reported improvements in dizziness-related disability and postural control following VBRT (124, 125), although the number of studies remains limited and intervention protocols and study designs are heterogeneous (126). TMS remains exploratory (127, 128).

### 5.1 Pharmacological treatment

Pharmacological treatment primarily includes SSRIs, SNRIs, BZDs, antihistamines, and other antidepressants.

#### 5.1.1 Selective serotonin reuptake inhibitors

SSRIs are used to treat PPPD (121, 129) and its precursor disorders (130–132). Representative agents include sertraline, paroxetine, fluvoxamine, escitalopram, and fluoxetine (121, 129–132). SSRIs primarily inhibit 5-HT reuptake mediated by the presynaptic serotonin transporter (SERT), thereby prolonging 5-HT action in the synaptic cleft and increasing serotonergic neurotransmission (133). Although selectivity varies among agents, SSRIs generally show greater affinity for SERT than for norepinephrine and dopamine transporters (133). Serotonergic neurons in the dorsal raphe nucleus project to both the vestibular nuclei and central amygdala (61), providing an anatomical basis for interactions between vestibular and anxiety-related circuits; however, the mechanisms by which SSRIs affect chronic dizziness remain uncertain. An uncontrolled case series reported improvement in dizziness and psychiatric symptoms after SSRI treatment (130), and a prospective open-label study of sertraline found reductions in Dizziness Handicap Inventory (DHI) scores in patients with CSD (131).

Fluoxetine has also been investigated in patients with dizziness and vestibular dysfunction (134). In addition to its serotonergic effects, experimental studies suggest that fluoxetine can inhibit K^+^ and transient inward Na^+^ currents in peripheral vestibular calyx afferent terminals, potentially reducing vestibular afferent excitability (135). However, the clinical relevance of these peripheral electrophysiological effects remains uncertain. Most available clinical evidence for SSRIs in chronic dizziness or PPPD derives from open-label, observational, retrospective, or precursor-disorder studies, and placebo-controlled trials specifically designed for PPPD remain lacking. Therefore, SSRIs should be regarded as commonly used treatment options supported by preliminary clinical evidence rather than PPPD-specific therapies with fully established efficacy.

#### 5.1.2 Serotonin-norepinephrine reuptake inhibitors

Representative SNRIs include venlafaxine, duloxetine, and milnacipran (136). SNRIs enhance serotonergic and noradrenergic neurotransmission by inhibiting the reuptake of 5-HT and NE, although their relative selectivity differs among agents (133, 136). Noradrenergic projections from the locus coeruleus to the vestibular nuclei have been demonstrated in animal studies (114), but their relationship to excessive monitoring of posture and movement in patients with PPPD remains uncertain. In a retrospective study by Yagi et al., DHI, Hospital Anxiety and Depression Scale (HADS), and Niigata PPPD Questionnaire (NPQ) scores improved in patients with PPPD treated with venlafaxine, escitalopram, mirtazapine, vortioxetine, or paroxetine (121). An earlier clinical study also reported improvement in DHI scores after treatment with escitalopram, venlafaxine, or mirtazapine (129). However, neither study used a placebo control; therefore, the specific efficacy of SNRIs cannot be confirmed from the available evidence.

#### 5.1.3 Benzodiazepines

BZDs include diazepam, lorazepam, clonazepam, and alprazolam. They act primarily as positive allosteric modulators of GABA_A_ receptors and enhance GABAergic inhibitory neurotransmission (8). In a randomized trial of acute vertigo, lorazepam reduced vertigo symptoms, but dimenhydrinate produced greater improvement and less sedation (137); a subsequent systematic review similarly found that single-dose antihistamines were more effective than BZDs for short-term relief (87). However, PPPD is a chronic functional vestibular disorder, and BZD-specific evidence in PPPD is lacking. In an uncontrolled case series of patients with chronic dizziness, prior treatment with BZDs provided only transient or incomplete relief, and sedation or cognitive impairment limited dose escalation; SSRIs were associated with greater improvement, although the study was not designed as a direct comparison (130). In older adults with dizziness, prescriptions for anxiolytic vestibular suppressants, including BZDs, were associated with a higher risk of medically attended falls (138). Prolonged BZD use may also lead to dependence and withdrawal symptoms (139). Therefore, available evidence does not support routine long-term use of BZDs for PPPD; if they are required for severe acute vertigo or anxiety, they should be used cautiously and for a limited duration.

#### 5.1.4 Antihistamines

Antihistamines used as vestibular suppressants include meclizine, dimenhydrinate, diphenhydramine, and promethazine. These first-generation agents have histamine H1 receptor antagonist and anticholinergic effects and are used mainly for the short-term control of acute vertigo, nausea, and motion sickness (8). A systematic review and meta-analysis found that single-dose antihistamines produced greater improvement in acute vertigo severity at approximately 2 h than single-dose BZDs. However, low-quality evidence did not show that daily antihistamine treatment was superior to placebo at 1 week or 1 month, and the included studies did not specifically evaluate PPPD (87).

Evidence concerning the interaction between vestibular suppressants and vestibular rehabilitation remains limited. In patients with chronic peripheral vestibular disorders, daily meclizine or diazepam for 6 weeks reduced dizziness but did not improve postural sway, whereas vestibular rehabilitation improved both dizziness and balance (140). However, the medication group was small and included both meclizine and diazepam. Another randomized study found that 2 weeks of cinnarizine–dimenhydrinate treatment did not reduce the efficacy of individualized vibrotactile neurofeedback training (141). These studies involved different vestibular disorders, medications, and rehabilitation protocols and do not establish that antihistamines impair vestibular rehabilitation in patients with PPPD.

First-generation antihistamines may cause sedation and anticholinergic cognitive adverse effects, particularly in older adults (8). In a prospective cohort of hospitalized patients aged 70 years or older, diphenhydramine exposure was associated with an increased risk of delirium symptoms, including inattention and altered consciousness (142). Because the long-term efficacy of antihistamines for PPPD has not been established (4) and clinically relevant adverse effects may occur, current evidence does not support their routine long-term use for PPPD. Brief use may be considered when severe acute vertigo, nausea, or motion sickness requires symptomatic control.

#### 5.1.5 Other antidepressants

Other antidepressants have also been used in observational studies of PPPD. Mirtazapine is a noradrenergic and specific serotonergic antidepressant (129), whereas vortioxetine is a multimodal serotonergic antidepressant (121). In a retrospective study of 43 patients with PPPD, three received mirtazapine and five received vortioxetine, but treatment outcomes were not analyzed separately for each drug (121). In another retrospective study, the mirtazapine group was too small for statistical efficacy analysis (129). No placebo-controlled randomized trial meeting the review criteria has established the efficacy of pharmacological treatment for PPPD (4). Therefore, current evidence is insufficient to support mirtazapine or vortioxetine as standard first-line treatments for PPPD.

Overall, current pharmacological approaches to PPPD can be summarized in three categories. The first comprises serotonergic and noradrenergic antidepressants, represented by SSRIs and SNRIs, which are commonly used in clinical practice, although high-quality placebo-controlled evidence remains lacking (4, 121, 129). The second category comprises vestibular suppressants, such as BZDs and first-generation antihistamines, which are used mainly for short-term control of acute symptoms (87, 137). Their routine long-term use for PPPD is not supported because PPPD-specific long-term efficacy has not been established and clinically relevant adverse effects may occur (4, 142). A third, exploratory category includes pharmacological targets related to glutamatergic and GABAergic signaling (23, 143), histaminergic and cholinergic signaling (31, 102), and dopaminergic and ion-channel mechanisms (118, 135). These targets have mainly been investigated in animal or *in vitro* vestibular models rather than in patients with PPPD and should therefore be regarded as mechanistic or exploratory targets rather than established treatments.

Long-term observational data from Niigata University Medical and Dental Hospital showed that DHI, HADS, and NPQ scores decreased from 3 months onward in patients with PPPD who continued antidepressant treatment; however, only 10 of 43 patients (23.3%) completely discontinued medication because their symptoms improved (121). Higher pretreatment DHI scores were associated with treatment response, defined as a decrease of at least 18 points, whereas pretreatment HADS scores were not associated with response (121). In a prospective uncontrolled study of 47 patients with chronic dizziness, improvements in subjective dizziness-related handicaps after paroxetine treatment were observed mainly in patients with high Zung Self-Rating Depression Scale scores, while no improvement was observed in those with low scores (144). These findings suggest that baseline symptom severity and psychological status may be associated with treatment response, but the available observational evidence is insufficient to recommend antidepressant therapy solely on the basis of high DHI scores or psychiatric comorbidity.

Vestibular pharmacology has largely relied on receptor-target studies and *in vitro* or ex vivo experimental models to characterize drug effects on vestibular sensory signaling (7, 94). Direct *in vivo* assessment remains important for determining how candidate agents affect vestibular function in intact organisms (7, 145).

### 5.2 Nonpharmacological treatments

Nonpharmacological treatments are an important component of the comprehensive management of PPPD, primarily including VBRT and CBT, with TMS serving as an exploratory adjunctive treatment (120).

#### 5.2.1 Vestibular and balance rehabilitation therapy

VBRT is an exercise-based intervention that primarily includes movement, postural-control, gaze-stabilization, sensory-substitution, and habituation exercises. It aims to promote vestibular adaptation, sensory substitution, and central compensation (146, 147). Thompson et al. conducted a retrospective chart review and telephone follow-up of patients with PPPD alone or PPPD with neurotological comorbidities after instruction in home-based VBRT. At follow-up, visual sensitivity and head-motion sensitivity were rated on separate 0–10 scales, while functional limitation was assessed using a 0–5 disability scale. Fourteen of 26 participants considered the exercises helpful, with some reporting relief of sensitivity to head/body motion or visual stimuli (148). Nada et al. (124) evaluated individualized vestibular rehabilitation using the DHI and found significant reductions in the total, functional, and physical scores after treatment. Ibrahim et al. assessed outcomes using the DHI and Sensory Organization Test (SOT) before and after 6 weeks of vestibular rehabilitation and reported improvements in dizziness-related disability and postural control (125). Anxiety and depression were assessed using the HADS, and improvements in SOT global scores were smaller in patients with abnormal anxiety or depression scores than in those with normal scores (125). These findings suggest that baseline anxiety and depression symptoms may be associated with postural-control responses to rehabilitation, although this result was derived from a small uncontrolled study. Two recent systematic reviews and meta-analyses reported significant reductions in DHI scores following vestibular rehabilitation; however, substantial heterogeneity and methodological limitations across the included studies limited the interpretation of the pooled findings (126, 149). High-quality randomized controlled evidence for VBRT in PPPD therefore remains limited (120, 126, 149). VBRT may engage activity-dependent neural plasticity and central vestibular compensation; however, existing studies in patients with PPPD have not directly evaluated its effects on neurotransmitter systems. Therefore, its relationship with neurotransmitter regulatory networks remains indirect. Treatment should be individualized according to symptom severity, comorbidities, adherence, and treatment response (147, 148).

#### 5.2.2 Cognitive behavioral therapy

CBT is a widely used psychosocial intervention in mental health care (150). CBT for chronic dizziness has included psychoeducation, behavioral experiments, exposure to feared stimuli, and attentional refocusing (151). CBT has no established direct neurotransmitter target. Small positron emission tomography studies in social anxiety disorder associated symptom improvement after CBT with changes in extrastriatal dopamine D2 receptor binding (152) and identified changes in serotonin and dopamine transporter availability during internet-delivered CBT administered with placebo or escitalopram (153). However, these findings provide only indirect evidence and do not establish a PPPD-specific neurotransmitter mechanism.

In a randomized controlled trial of patients with CSD, CBT reduced dizziness-related symptoms, disability, and avoidance and safety behaviors but did not significantly change depression, anxiety, or stress scores (151). In a retrospective single-group study of 150 patients with PPPD, CBT was associated with pre-to-post reductions in dizziness symptoms, dizziness-related disability, avoidance and safety behaviors, and anxiety (10). CBT has also been investigated as an adjunct to pharmacotherapy. In an 8-week randomized controlled trial, compared with sertraline alone, CBT combined with sertraline produced greater reductions in DHI, anxiety, and depression scores, while requiring lower sertraline doses and resulting in fewer adverse events (122). Observational studies have also reported symptomatic improvement after multimodal programs integrating CBT, vestibular rehabilitation, and serotonergic medication when indicated (154, 155). However, these uncontrolled designs do not permit the effects of the individual treatment components to be distinguished. A meta-analysis of six randomized controlled trials involving patients with PPPD or CSD found that adding CBT to vestibular rehabilitation, a selective serotonin reuptake inhibitor, or their combination improved DHI and anxiety- and depression-related scores compared with these treatments alone (123). However, only three included trials enrolled patients with PPPD, and the interventions and control treatments varied across studies. Further high-quality randomized controlled trials are therefore needed. CBT may be considered a component of comprehensive treatment for PPPD, particularly when anxiety or avoidance behaviors are treatment targets.

#### 5.2.3 Transcranial magnetic stimulation

In addition, TMS is an exploratory approach for the treatment of PPPD. TMS induces cortical currents through time-varying magnetic fields and can influence excitability in local cortical regions and connected brain networks (156). Repetitive transcranial magnetic stimulation (rTMS), a form of TMS that delivers magnetic pulses repeatedly, may produce neuromodulatory effects that outlast the stimulation period (157). These effects may partly involve neurotransmitter systems. In patients with treatment-resistant depression, rTMS was associated with increased GABA levels at the left dorsolateral prefrontal cortex stimulation site (158). In healthy volunteers, prefrontal rTMS induced endogenous dopamine release in the ipsilateral caudate nucleus (159). However, these findings were obtained in non-PPPD populations. Direct evidence that TMS alters neurotransmitter function in patients with PPPD is currently lacking. Thus, neurotransmitter modulation is a plausible but unconfirmed mechanism of TMS in PPPD.

Imaging studies of PPPD suggest structural or functional changes in regions involved in vestibular processing, multisensory integration, visual processing, postural control, and emotional regulation (3, 160). These findings provide a theoretical basis for neuromodulation therapies. The available PPPD-specific rTMS trials have targeted the left dorsolateral prefrontal cortex (127, 128). This target may be relevant to cognitive control and emotional regulation, which are implicated in PPPD (160). In a randomized, double-blind, sham-controlled trial, Jia et al. compared active rTMS with sham rTMS as an adjunct to conventional treatment. The conventional treatment included vestibular rehabilitation therapy and maintenance therapy with a SSRI or SNRI (127). After 4 weeks, DHI, Hamilton Anxiety Rating Scale, and Hamilton Depression Rating Scale scores were lower in the active-rTMS group than in the sham-rTMS group (127). These results suggest a potential adjunctive effect on dizziness-related disability, anxiety, and depressive symptoms. Li et al. conducted a single-center, single-blind, randomized, sham-controlled trial of high-frequency TMS over the left dorsolateral prefrontal cortex (128). At 2 weeks, 1 month, and 3 months after treatment, dizziness and anxiety scores were lower in the TMS group than at baseline and in the sham-stimulation group (128). No serious adverse events were reported (128). These findings suggest that TMS may serve as a complementary approach in the comprehensive treatment of PPPD. However, the current evidence base remains limited, and the optimal stimulation target, frequency, treatment course, maintenance regimen, and long-term efficacy remain uncertain (161). Larger multicenter studies with longer follow-up are needed to confirm its efficacy, safety, and mechanisms of action (127, 128, 161). At present, TMS should not be considered a routine first-line treatment for PPPD. It may be considered only as an exploratory adjunct for carefully selected patients or in clinical research settings.

Table 2 provides a concise summary of therapeutic options for PPPD and their current level of supporting evidence. The detailed therapeutic evidence framework is presented in Supplementary Table 1.

**Table 2:** Therapeutic options for persistent postural-perceptual dizziness and level of supporting evidence.

Intervention	Current evidence	Main limitations	Level of evidence	Clinical positioning	Key references
Selective serotonin reuptake inhibitors	These agents are widely used in clinical practice. The evidence mainly derives from open-label studies, observational studies, retrospective studies, or studies of CSD.	High-quality placebo-controlled randomized trials specifically targeting PPPD are lacking.	Low	Commonly used pharmacological options in clinical practice, but they should not yet be regarded as fully established disease-specific treatments.	(4, 121, 129–132, 134, 144)
Serotonin–norepinephrine reuptake inhibitors	The evidence mainly comes from observational or retrospective studies, with some data from antidepressant treatment studies in PPPD.	PPPD-specific placebo-controlled trials are lacking, and evidence for stratified treatment remains limited.	Low	These agents may be considered individualized treatment options for selected patients.	(4, 121, 129)
Benzodiazepines	The evidence mainly relates to acute vertigo or short-term symptom control.	They may increase the risks of sedation, cognitive impairment, falls, dependence, and withdrawal symptoms. Evidence regarding their effects on central vestibular compensation in PPPD remains limited.	Low evidence for long-term treatment of PPPD	They are not recommended as routine long-term therapy for PPPD and should be used only briefly and cautiously when necessary.	(8, 87, 88, 137–139)
Antihistamines	Evidence from patients with acute vertigo suggests short-term symptomatic benefit.	These findings cannot be directly extrapolated to PPPD. Long-term efficacy in PPPD has not been established, and first-generation agents may cause sedation and anticholinergic cognitive adverse effects.	Low evidence for long-term treatment of PPPD	They are not suitable as core long-term therapy for PPPD.	(4, 8, 87, 140–142)
Other antidepressants	Evidence is limited to small-sample or observational studies.	PPPD-specific evidence is limited and is insufficient to support these agents as standard first-line treatments for PPPD.	Low	They may be considered individualized options for patients with specific comorbidities.	(4, 121, 129)
Vestibular and balance rehabilitation therapy	Observational studies and meta-analyses suggest improvements in DHI scores and related outcomes.	Intervention protocols are highly heterogeneous, and high-quality randomized controlled studies specifically addressing PPPD remain limited.	Moderate, with substantial heterogeneity	A core component of individualized rehabilitation management.	(120, 124–126, 146–149)
Cognitive behavioral therapy	Randomized controlled studies and meta-analyses suggest potential benefit as an adjunctive treatment.	The number of studies remains limited, and further high-quality randomized controlled trials are needed.	Moderate	An important component of comprehensive PPPD management.	(10, 122, 123, 151, 154, 155)
Transcranial magnetic stimulation	Evidence comes from small randomized sham-controlled rTMS studies and limited non-pharmacological intervention data.	Sample sizes are small, most studies are single-center, and long-term efficacy, optimal targets, and stimulation protocols remain unclear.	Exploratory	TMS may be considered an investigational or carefully selected adjunctive therapy, but it should not be regarded as routine first-line treatment.	(127, 128, 161)

PPPD, persistent postural-perceptual dizziness; SSRIs, selective serotonin reuptake inhibitors; SNRIs, serotonin–norepinephrine reuptake inhibitors; 5-HT, 5-hydroxytryptamine; NE, norepinephrine; GABA, gamma-aminobutyric acid; CSD, chronic subjective dizziness; DHI, Dizziness Handicap Inventory; VBRT, vestibular and balance rehabilitation therapy; CBT, cognitive behavioral therapy; TMS, transcranial magnetic stimulation; rTMS, repetitive transcranial magnetic stimulation.

The levels of evidence represent a narrative classification used in this review based on study design, sample size, comparison methods, consistency of findings, and specificity for PPPD; they do not constitute a formal GRADE assessment. “High” indicates support from multiple high-quality randomized controlled trials or high-quality systematic reviews; “Moderate” indicates support from randomized trials, meta-analyses, or relatively consistent clinical studies, although limitations remain regarding sample size, heterogeneity, comparison methods, or follow-up duration; “Low” indicates that evidence primarily comes from observational studies, open-label studies, retrospective studies, case series, or studies of conditions preceding PPPD; “Exploratory” indicates that the evidence primarily comes from small-sample, single-center, mechanistic studies, animal or in vitro studies, or early exploratory clinical trials.

## 6 Discussion

A major limitation of PPPD research is the absence of validated objective biomarkers. Current diagnosis relies primarily on clinical symptoms, temporal characteristics, exacerbating factors, and the assessment of other active disorders (1). No validated blood, cerebrospinal fluid, imaging, or neurotransmitter biomarker is currently available for routine diagnosis. Future research should establish prospective multicenter cohorts and integrate symptom scales, vestibular function tests, measures of postural control and visual dependence, psychological and behavioral indicators, and neuroimaging measures. This multidimensional approach may improve the characterization of PPPD heterogeneity.

Neurotransmitter-related imaging is an important direction for future research. Existing PPPD imaging studies have primarily used structural magnetic resonance imaging (MRI) (162), resting-state functional magnetic resonance imaging (fMRI) (163), and task-based fMRI (13). However, studies using positron emission tomography (PET) to assess serotonergic, noradrenergic, or dopaminergic targets, or magnetic resonance spectroscopy (MRS) to quantify GABA- and Glu-related metabolites, remain lacking (3, 160). Future studies could combine PET or MRS with task-based fMRI and vestibular stimulation to examine associations between neurochemical measures and visual dependence, postural hypervigilance, anxiety responses, and treatment outcomes. Such studies may help distinguish PPPD-related network alterations from changes associated with comorbid anxiety, depression, or residual vestibular dysfunction.

A single case–control study involving 43 patients with PPPD and 45 controls reported a higher frequency of the A1 allele of the *DRD2*/*ANKK1* TaqIA polymorphism (rs1800497) in the PPPD group, suggesting a possible association with susceptibility to PPPD (66). This finding requires replication in larger multicenter cohorts that include diverse populations. Future analyses should also incorporate personality traits, anxiety severity, vestibular triggers, and treatment responses. Beyond candidate gene studies, genome-wide association studies, epigenetic analyses, and gene–environment interaction studies may help clarify the heterogeneity of PPPD and the risk of chronicity.

Metabolomics and the gut-brain axis remain exploratory research directions. Human-derived intestinal Bacteroides strains have been shown to produce GABA *in vitro* (164). A six-week aerobic exercise intervention also altered gut microbiota composition and function in previously sedentary adults without PPPD (165). However, these findings cannot be directly extrapolated to PPPD. There is currently insufficient evidence to establish gut microbiota or metabolomic abnormalities as causes of PPPD or validated therapeutic targets. Accordingly, gut microbiome and metabolomic studies should currently be framed as exploratory approaches to biomarker screening and subtyping rather than as evidence of an established PPPD mechanism.

In terms of clinical research, further multicenter randomized controlled trials (RCTs) are needed (4). Future pharmacological trials should use standardized PPPD diagnostic criteria and consider stratified enrollment. They should clearly define eligibility criteria and comorbidities and report dosage, treatment duration, adverse events, and discontinuation strategies. Appropriate placebo controls and adequate long-term follow-up should also be included. Non-pharmacological studies should standardize intervention protocols, treatment intensity, concomitant treatments, and follow-up schedules for CBT (122), VBRT (149, 166), and TMS (127, 128). Outcome measures should include the DHI, NPQ, HADS, measures of postural control and visual motion sensitivity, quality of life, adverse events, and long-term symptom recurrence. Consistent outcome sets would facilitate comparisons across treatment modalities. Future PPPD research should evaluate stratified treatment models based primarily on symptom phenotypes and comorbidities, while examining whether neuroimaging characteristics provide additional predictive value.

In summary, the molecular mechanisms underlying PPPD remain unclear. Current evidence is derived mainly from basic vestibular studies, neuroimaging research, studies of chronic dizziness, and a limited number of PPPD-specific clinical studies. Diagnosis continues to rely on clinical criteria and the assessment of other active disorders because validated objective biomarkers are lacking (1). Current evidence does not establish a single treatment approach suitable for all patients. Placebo-controlled evidence for SSRIs and SNRIs remains unavailable (4), and PPPD-specific rTMS evidence is limited to small single-center trials (127, 128). Future multicenter trials with appropriate control groups and long-term follow-up should evaluate efficacy, safety, treatment-response subgroups, and multimodal treatment models. Further mechanistic studies may identify new therapeutic targets. Although the neurotransmitter mechanisms discussed in this review are biologically plausible, most are not supported by direct PPPD-specific evidence. Findings from animal experiments, ex vivo studies, and studies of other vestibular disorders should therefore not be interpreted as direct evidence of clinical mechanisms in PPPD.

## 7 Conclusion

Although different patients may present with similar dizziness or vestibular symptoms, their rehabilitation trajectories and prognostic outcomes can differ significantly. These differences may be attributed to interactions among dizziness-associated disorders, where multiple factors reinforce one another, creating a maladaptive cycle that diminishes treatment response and exacerbates prognosis. To address these clinical challenges, this review examines potential neurotransmitter-related mechanisms in PPPD and provides a neuropharmacological basis for pharmacological interventions and individualized rehabilitation strategies. Neurotransmitter networks present a promising framework for understanding the persistence of PPPD symptoms, the comorbidity of anxiety, and treatment responses; however, the long-term efficacy of novel targeted therapies necessitates validation through multicenter RCTs.

At present, PPPD cannot be explained as a disorder caused by a single neurotransmitter abnormality. Systems involving 5-HT, NE, DA, GABA, Glu, and histamine may participate in vestibular regulation, anxiety comorbidity, and treatment response, but their PPPD-specific roles require further validation. Future research should employ standardized diagnostic criteria, integrate neurotransmitter-related neuroimaging, utilize genetic and metabolomic approaches, and conduct multicenter clinical trials to establish more reliable phenotyping and individualized treatment strategies.
