# Supplementary material for: Neurotransmitter regulatory networks in persistent postural-perceptual dizziness: potential mechanisms and therapeutic implications
Source: Front Neurol. 2026 Sep 8;17:1921702. doi: 10.3389/fneur.2026.1921702 (PMC13595364; doi:10.3389/fneur.2026.1921702)
Supplement: Supplementary file 1 [file Table_1.pdf]

# Supplementary Material

## 1 Supplementary Table

**Supplementary Table 1. Detailed therapeutic options for persistent postural-perceptual dizziness and level of supporting evidence.**

| Intervention | Category | Representative agents or intervention components | Proposed mechanism or therapeutic target | Applicable population or clinical phenotype | Current evidence | Main limitations | Level of evidence | Clinical positioning | Key references |
|---|---|---|---|---|---|---|---|---|---|
| Selective serotonin reuptake inhibitors | Antidepressants | Sertraline, paroxetine, fluvoxamine, escitalopram, fluoxetine, and others | These agents mainly inhibit serotonin transporter-mediated presynaptic 5-HT reuptake, thereby enhancing serotonergic neurotransmission. Serotonergic vestibular–affective circuits provide a possible rationale, but the mechanisms by which SSRIs affect chronic dizziness remain uncertain. | Patients with PPPD considered for individualized pharmacotherapy, including those with comorbid anxiety or depressive symptoms; no validated response-predictive phenotype has been established. | These agents are widely used in clinical practice. The evidence mainly derives from open-label studies, observational studies, retrospective studies, or studies of CSD. | High-quality placebo-controlled randomized trials specifically targeting PPPD are lacking. | Low | Commonly used pharmacological options in clinical practice, but they should not yet be regarded as fully established disease-specific treatments. | (4, 121, 129–132, 134, 144) |
| Serotonin–norepinephrine reuptake inhibitors | Antidepressants | Venlafaxine, duloxetine, milnacipran, and others | These agents inhibit 5-HT and NE reuptake and enhance serotonergic and noradrenergic neurotransmission. Noradrenergic pathways are involved in vigilance, arousal, stress responses, and vestibular regulation, but their relationship to postural hypervigilance in PPPD remains uncertain. | Selected patients with PPPD, including those with comorbid anxiety or depressive symptoms; evidence does not establish a response-predictive phenotype. | The evidence mainly comes from observational or retrospective studies, with some data from antidepressant treatment studies in PPPD. | PPPD-specific placebo-controlled trials are lacking, and evidence for stratified treatment remains limited. | Low | These agents may be considered individualized treatment options for selected patients. | (4, 121, 129) |
| Benzodiazepines | Sedative–hypnotic and anxiolytic agents | Diazepam, lorazepam, clonazepam, alprazolam, and others | These agents enhance inhibitory neurotransmission through positive allosteric modulation of $GABA_A$ receptors and may transiently reduce vestibular excitability or acute anxiety. | Patients with acute vertigo, marked acute anxiety, or short-term acute symptom control needs. | The evidence mainly relates to acute vertigo or short-term symptom control. | They may increase the risks of sedation, cognitive impairment, falls, dependence, and withdrawal symptoms. Evidence regarding their effects on central vestibular compensation in PPPD remains limited. | Low evidence for long-term treatment of PPPD | They are not recommended as routine long-term therapy for PPPD and should be used only briefly and cautiously when necessary. | (8, 87, 88, 137–139) |
| Antihistamines | Vestibular suppressants | Meclizine, dimenhydrinate, promethazine, and diphenhydramine | These agents have H1 receptor antagonistic and anticholinergic effects and may provide short-term relief of acute vertigo, nausea, or motion sickness. | Patients with acute vertigo, nausea, vomiting, or motion sickness. | Evidence from patients with acute vertigo suggests short-term symptomatic benefit. | These findings cannot be directly extrapolated to PPPD. Long-term efficacy in PPPD has not been established, and first-generation agents may cause sedation and anticholinergic cognitive adverse effects. | Low evidence for long-term treatment of PPPD | They are not suitable as core long-term therapy for PPPD. | (4,8,87,140–142) |

| Intervention | Category | Representative agents or intervention components | Proposed mechanism or therapeutic target | Applicable population or clinical phenotype | Current evidence | Main limitations | Level of evidence | Clinical positioning | Key references |
|---|---|---|---|---|---|---|---|---|---|
| Other antidepressants | Antidepressants | Mirtazapine, vortioxetine, and others | Multimodal 5-HTergic or noradrenergic modulation. | Patients with sleep disturbance, anxiety, depressive symptoms, or intolerance to SSRIs/SNRIs. | Evidence is limited to small-sample or observational studies. | PPPD-specific evidence is limited and is insufficient to support these agents as standard first-line treatments for PPPD. | Low | They may be considered individualized options for patients with specific comorbidities. | (4,121,129) |
| Vestibular and balance rehabilitation therapy | Rehabilitation therapy | Movement, postural-control, gaze-stabilization, sensory-substitution, and habituation exercises. | VBRT promotes vestibular adaptation, sensory substitution, and central compensation. | Patients with visual motion sensitivity, motion sensitivity, postural instability, or functional impairment. | Observational studies and meta-analyses suggest improvements in DHI scores and related outcomes. | Intervention protocols are highly heterogeneous, and high-quality randomized controlled studies specifically addressing PPPD remain limited. | Moderate, with substantial heterogeneity | A core component of individualized rehabilitation management. | (120,124–126,146–149) |
| Cognitive behavioral therapy | Psychological intervention | Psychoeducation, behavioral experiments, exposure to feared stimuli, and attentional refocusing. | CBT may improve hypervigilance, avoidance behavior, maladaptive cognition, and anxiety-related symptoms. | Patients with anxiety, avoidance behavior, or illness-related hypervigilance. | Randomized controlled studies and meta-analyses suggest potential benefit as an adjunctive treatment. | The number of studies remains limited, and further high-quality randomized controlled trials are needed. | Moderate | An important component of comprehensive PPPD management. | (10, 122, 123, 151, 154, 155) |
| Transcranial magnetic stimulation | Neuromodulation | High-frequency rTMS over the left dorsolateral prefrontal cortex. | TMS may modulate local cortical excitability and connected brain networks. Studies in non-PPPD populations suggest possible effects on GABA and dopamine, but direct evidence of neurotransmitter modulation in PPPD is lacking. | Carefully selected patients as an exploratory adjunct or patients treated in clinical research settings. | Evidence comes from small randomized sham-controlled rTMS studies and limited non-pharmacological intervention data. | Sample sizes are small, most studies are single-center, and long-term efficacy, optimal targets, and stimulation protocols remain unclear. | Exploratory | TMS may be considered an investigational or carefully selected adjunctive therapy, but it should not be regarded as routine first-line treatment. | (127, 128, 161) |

Abbreviations: PPPD, persistent postural-perceptual dizziness; SSRIs, selective serotonin reuptake inhibitors; SNRIs, serotonin–norepinephrine reuptake inhibitors; 5-HT, 5-hydroxytryptamine; NE, norepinephrine; GABA, gamma-aminobutyric acid; CSD, chronic subjective dizziness; DHI, Dizziness Handicap Inventory; VBRT, vestibular and balance rehabilitation therapy; CBT, cognitive behavioral therapy; TMS, transcranial magnetic stimulation; rTMS, repetitive transcranial magnetic stimulation.

Note: The levels of evidence represent a narrative classification used in this review based on study design, sample size, comparison methods, consistency of findings, and specificity for PPPD; they do not constitute a formal GRADE assessment. “High” indicates support from multiple high-quality randomized controlled trials or high-quality systematic reviews; “Moderate” indicates support from randomized trials, meta-analyses, or relatively consistent clinical studies, although limitations remain regarding sample size, heterogeneity, comparison methods, or follow-up duration; “Low” indicates that evidence primarily comes from observational studies, open-label studies, retrospective studies, case series, or studies of conditions preceding PPPD; “Exploratory” indicates that the evidence primarily comes from small-sample, single-center, mechanistic studies, animal or in vitro studies, or early exploratory clinical trials.
